# Effectiveness of Jian-Pi-An-Tai formula for the pregnancy outcome of in vitro fertilization and embryo transfer in infertile women: Protocol of a randomized controlled trial

**DOI:** 10.1097/MD.0000000000032419

**Published:** 2022-12-23

**Authors:** Ran Cheng, Qing Liu, Ying Zhu, Ying Zhao, Liuqing Yang, Qin Zhang

**Affiliations:** a Department of Traditional Chinese Medical Gynecology, Hangzhou TCM Hospital Affiliated to Zhejiang Chinese Medical University, Hangzhou, China; b School of Basic Medical Sciences, Zhejiang Chinese Medical University, Hangzhou, China.

**Keywords:** in vitro fertilization and embryo transfer, infertility, Jian-, Pi-An-Tai formula, randomized controlled trial

## Abstract

Infertility has become a global public health issue. In vitro fertilization and embryo transfer (IVF-ET) is widely performed as an infertility treatment. However, a significant number of infertile women continue to experience serial implantation failure, despite the high quality of the transferred embryos. Jian-Pi-An-Tai formula is an experience formula in treating threatened abortion in our team. It has also been used to treat infertile women after embryo transfer and shows superiority compared to single use of conventional western medicine. However, the of evidence on its effective in treating infertile women undergo IVF-ET is lacking.

**Methods:**

This randomized controlled trial (RCT) will be carried out in Hangzhou TCM Hospital Affiliated to Zhejiang Chinese Medical University planning to recruit 180 infertile patients undergoing IVF-ET with the type of deficiency of both the spleen and kidney. The control group will be treated by conventional western medicine and the treatment group will use conventional western medicine plus Jian-Pi-An-Tai formula. The primary outcomes will include Embryo implantation rate, Clinical pregnancy rate, Persistent pregnancy rate; and the secondary outcomes will include TCM symptom score and reproductive hormones. Safety evaluation will be recorded during the whole study. All data in this RCT will be analyzed by SPSS 23.0 software. This study has been approved by the Research Ethics Committee of the Hangzhou TCM Hospital Affiliated to Zhejiang Chinese Medical University (2022KY130).

**Discussion:**

The results of this RCT will contribute to provide scientific and rigorous evidence for the efficacy and safety of Jian-Pi-An-Tai formula in treating infertile women undergo IVF-ET. And the results from this RCT will be published in a relevant journal after finished.

## 1. Introduction

Couples who have a normal sexual life but do not conceive naturally for more than a year are considered to have infertility. Infertility has become the third most serious disease, making it a global public health issue.^[1]^ Numerous factors, including hormonal imbalance, genetics, diet, smoking, caffeine use, and alcohol consumption, are linked to infertility. In vitro fertilization and embryo transfer (IVF-ET) is widely performed as an infertility treatment worldwide, resulting in the births of more than 5 million infants worldwide.^[2]^ Despite the remarkable advancements in assisted reproductive technology (ART), a significant number of infertile women continue to experience serial implantation failure, despite the high quality of the transferred embryos. In addition, women conceived through IVF-ET have a higher rate of threatened abortions than natural pregnancy.^[3]^ It is crucial to improve the implantation rate and reduce the abortion rate after embryo transfer for the success of IVF-ET.

The process of embryo implantation, which involves both the embryo and the mother’s endometrium, is intricate. One of the main causes of unsuccessful embryo implantation is assumed to be impaired endometrial receptivity.^[4]^ A healthy pregnancy is built on a foundation of appropriate implantation processes, which are facilitated by optimal receptivity. Endocrine reasons, inflammatory events, thin endometria, fibroids, polyps, septa, and immunologically induced abnormalities are only a few of the contributing elements that affect receptivity. Although implantation failure in IVF-ET is a very common occurrence, there are currently no evidence-based therapeutic treatments available. Therefore, current therapy approaches are mostly empirical, based on some biologic justification, but have scant clinical proof to back them up.^[5,6]^

Chinese herbal medicines have been widely used in Asian countries for millennia to treat a variety of reproductive problems, and they hold promise for the future of human reproductive health, notably in the treatment of recurrent miscarriage and ART drugs.^[7]^Numerous clinical and experimental studies have demonstrated the effectiveness of the traditional Chinese medicine in the prevention and treatment of recurrent spontaneous abortion, in increasing the number and quality of high-quality embryos, in repairing endometrium, and in improving uterine receptivity.^[8–11]^ The spleen and kidney are the foundation of acquired constitution and congenital constitution respectively. Along with the spleen, the spleen governs transportation and transformation, generating qi and blood for the entire body. The main cause of the embryo’s failure to develop normally is deficiency of both the spleen and kidney.

Jian-Pi-An-Tai formula is an experience formula in treating threatened abortion (Deficiency of both the spleen and kidney type) for more than 50 years in our team. In recent years, Jian-Pi-An-Tai formula has also been used to treat infertile women after embryo transfer and shows superiority compared to single use of conventional western medicine. However, the of evidence on its effective in treating infertile women undergo IVF-ET is lacking. Therefore, the aim of this study is to verify the clinical efficacy and safety of Jian-Pi-An-Tai formula for the pregnancy outcome of IVF-ET in infertile women.

## 2. Methods

### 2.1. Trial design

**Figure 1.**
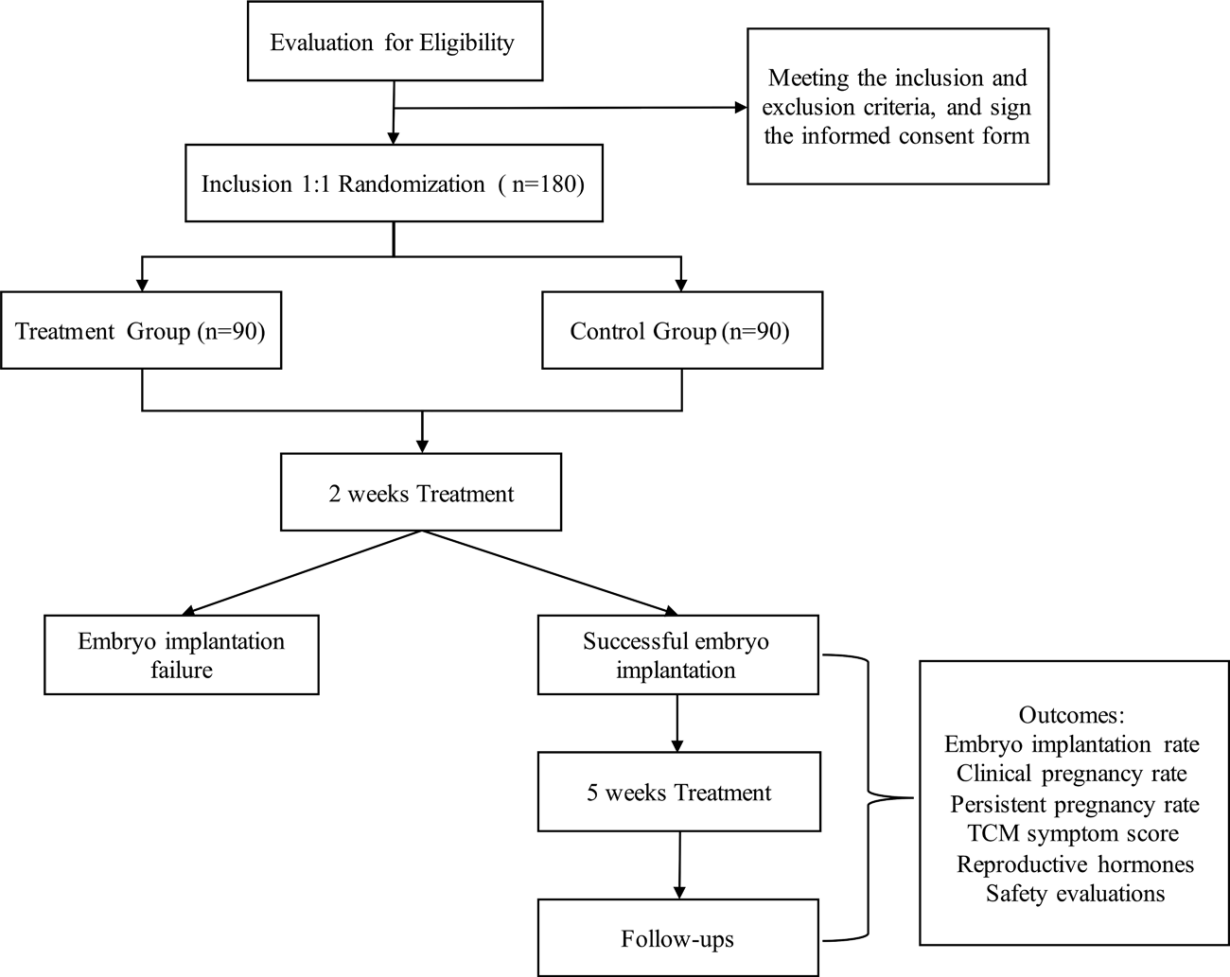
Study flow chart.

This study is a protocol for a randomized, non-blinded, controlled clinical trial. A total of 180 infertile patients with the type of Deficiency of both spleen and kidney will be conducted according to the flow diagram and will last for near 2 years, scheduled to start from January 2023 (Fig. 1).

### 2.2. Ethics committee

The study will follow the human research regulations of the Research Ethics Committee of the Hangzhou TCM Hospital Affiliated to Zhejiang Chinese Medical University, under the process number 2022KY130. After all aspects of participation in the research have been clarified, participants are required to sign a written informed consent form. One of the researchers responsible for the procedures will explain the consent forms. The study will be carried out in accordance with the Declaration of Helsinki. The study participants will be recruited at Run Run Shaw Hospital affiliated to Zhejiang University and treated at the Hangzhou TCM Hospital Affiliated to Zhejiang Chinese Medical University, from January 1, 2023 to October 31, 2024.

### 2.3. Participants

#### 2.3.1. Inclusion criteria.

1. Patients who meet the diagnostic criteria for infertility and spleen kidney deficiency syndrome;
2. Patients who plan to perform ART in the Reproductive Department of Run Run Shaw Hospital affiliated to Zhejiang University and plan to perform frozen embryo transfer; the transferred embryos are 2 superior embryos;
3. 25 to 40 years old;
4. Patients who have been insured in Hangzhou TCM Hospital Affiliated to Zhejiang Chinese Medical University after embryo transfer;
5. Signed informed consent.

#### 2.3.2. Exclusion criteria.

1. Patients with genital tract malformation or other reproductive system diseases (adenomyosis, endometrial tuberculosis, etc);
2. Suffering from sexually transmitted diseases or acute inflammation of urogenital system;
3. Patients with serious primary diseases such as cardiovascular, cerebrovascular, kidney, liver and hematopoietic system;
4. Patients with serious cognitive impairment and mental illness;
5. Long term exposure to toxic and harmful substances such as radiation or chemicals;
6. Incomplete data or unauthorized use of other treatments;
7. Patients who are allergic to the drugs used in this study.

### 2.4. Randomization and blinding

The randomization succession will be created with the PROC PLAN strategy explanations of SAS programming 9.3 bundle by the analyst specialists of Zhejiang Chinese Clinical College, and will be hidden and scattered utilizing obscure envelopes. During the time spent treating and analyzing the data, these specialists will not be involved. By phone, the clinical investigators will apply for a new included patient’s randomization number. The doctors and patients engaged in this RCT won’t likely be rendered blind because there is no usage of herbal medicine in the control group.

### 2.5. Interventions

The randomization strategy will be used to divide the participants into one of the 2 groups.

The treatment group: conventional western medicine after embryo transplantation combined with Jian-Pi-An-Tai formula. Jian-Pi-An-Tai formula is composed of codonopsis pilosula, astragalus membranaceus, fried atractylodes macrocephala, donkey hide gelatin, mulberry parasitic, dodder seed, raw rehmannia root, cooked rehmannia root, ramie root, scutellaria baicalensis, paeony root, wolfberry fruit, eclipta alba, perilla frutescens stem, licorice root, and dipsacus, which provided by Preparation Center of the Hangzhou TCM Hospital Affiliated to Zhejiang Chinese Medical University. The patients are planned to take Jian-Pi-An-Tai formula for 5 weeks.

The control group: conventional western medicine after embryo transplantation.

### 2.6. Outcome measures

#### 2.6.1. Primary outcome measures

1. Embryo implantation rate: number of embryos implanted/total number of embryos transferred * 100%. Blood sampling test of β-HCG will be taken on the 8th and 10th days after embryo transfer. Positive results can be determined as successful embryo implantation.
2. Clinical pregnancy rate: number of clinical pregnancies/number of implanted embryos * 100%. Doppler ultrasound will be taken on the 5th week after embryo transfer. Gestational sac and fetal heart beat can be determined as successful clinical pregnancy.
3. Persistent pregnancy rate: number of cycles of continuous pregnancy/number of embryos implanted * 100%. Doppler ultrasound will be taken on the 12th week after embryo transfer. Existence of fetal heart beat can be determined as successful persistent pregnancy.

#### 2.6.2. Secondary outcome measures.

##### 2.6.2.1. TCM symptom score.

The form of TCM symptom will be measured at the baseline, after 5 weeks of treatment.

##### 2.6.2.2. Reproductive hormone.

Blood sampling test of β-HCG, estradiol (E2), progesterone (P) levels will be measured once a week after embryo implantation, and additional tests should be conducted at any time according to the clinical needs.

### 2.7. Safety evaluation

Blood sampling test of liver function and blood routine examination will be conducted before, during, and after treatment of Jian-Pi-An-Tai formula.

### 2.8. Sample size calculation

The number of participants is capable to detecting according to the results of the previous study (overall response rate of was 86.2% in the treatment group; 54.2% in the control group) with an alpha error of 0.05 and beta error of 0.2. Considering the 10% drop-out rates, the sample will be composed of 180 participants randomized into 2 groups.

### 2.9. Statistical analysis

The statistical analysis of this study will be conducted by statisticians of the Zhejiang Chinese Medical University. Software packages SPSS 23.0 and SAS 9.3 will be used for statistical analysis. All statistical tests will be conducted by 2-sided tests and *P* values less than .05 are considered statistically significant. The description of quantitative indicators will calculate the number of cases, mean, standard deviation, median, minimum and maximum. Classification indicators are described by the number of cases and percentages of each category. Normally distributed data is represented by mean ± standard deviation, and non-normally distributed data is represented by median and interquartile range. Comparisons between groups of measurement data will be conducted by using independent sample *t* test, and within group differences will be tested with paired *t* test. Differences between groups of numeration data will be assessed with chi-square test.

## 3. Discussion

Over the past 10 years, the rate of infertility has significantly increased, making it a significant public health issue for women of reproductive age worldwide. Infertility brings families heavy economic and psychological burden, affecting social harmony and stability. In addition, a recent meta-analysis demonstrates that intimate partner violence against infertile women occurs frequently, particularly in low- and middle-income countries.^[12]^ An increasing number of families are opting for IVF-ET to get pregnancy. However, what we are facing today is that implantation failure and threatened abortion in IVF-ET is common, but we lack the evidence-based therapeutic solutions for treating it.

In China, the application of Chinese herbal medicines go through the process of IVF-ET, well known for its economical, safe and no side effects. Chinese herbal medicines shows a significant effect on improving endometrial receptivity, added to these are regulating hormone secretion, improving oocyte quality, relieving patients’ anxiety, and reducing inflammation. However, there is insufficient and poor-quality evidence to support its efficacy in treating infertile IVF-ET patients. Hence, randomized controlled trials are necessary to provide higher evidence for clinical use. The trial is valuable for it may provide scientific and rigorous evidence for the efficacy and safety of Jian-Pi-An-Tai formula in treating infertile women undergo IVF-ET.

## Author contributions

All authors read and critically revised the protocol.

**Conceptualization:** Qing Liu, Liuqing Yang, Qin Zhang.

**Data curation:** Ran Cheng, Qing Liu, Liuqing Yang.

**Formal analysis:** Ying Zhu, Ying Zhao.

**Investigation:** Qin Zhang.

**Methodology:** Ran Cheng, Liuqing Yang.

**Supervision:** Qin Zshang.

**Validation:** Yi-Zhe Zhang.

**Writing – original draft:** Ran Cheng, Qing Liu.

**Writing – review & editing:** Ran Cheng, Qing Liu, Liuqing Yang, Qin Zhang.
